# Supplementary material for: Measuring Myotonia: Normative Values and Comparison with Myotonic Dystrophy Type 1
Source: Neurol Int. 2025 Jul 31;17(8):118. doi: 10.3390/neurolint17080118 (PMC12389102; doi:10.3390/neurolint17080118)
Supplement: Supplementary file 1 [file neurolint-17-00118-s001.zip › neurolint-3653002-supplementary.pdf]

Article

Measuring Myotonia: Normative Values and Comparison with Myotonic Dystrophy Type 1

Andrea Sipos [1], Milán Árvai [1], Dávid Varga [1], Brigitta Ruszin-Perecz [1], József Janszky [1], Nándor Hajdú [2] and Endre Pál [1,*]

[1] Department of Neurology, University of Pécs, Medical School, 7623 Pécs, Hungary; sipos.andrea@aok.pte.hu (A.S.); arvaimilan11@gmail.com (M.Á.); varga.david@pte.hu (D.V.); brigittaperecz@gmail.com (B.R.-P.); janszky.jozsefd@pte.hu (J.J.)

[2] Institute of Psychology, Eötvös Loránd University,1053 Budapest, Hungary; hajdu.nandor93@gmail.com

* Correspondence: pal.endre@pte.hu

Supplementary Table S1. Average results of grip strength and functional tests by age group. Values originally predicted as negative by the linear model have been set to 0 kg, as negative handgrip strength is not biologically possible. This reflects the limitations of linear extrapolation in extreme demographic/clinical combinations.

Age	Group	Sex	Grip Strength, Dominant (kg)	Grip Strength Non-dominant (kg)	10 Eye Openings (s)	10 Palm Openings (s)	10 Sticking Tongue Out (s)	Nine-Hole Peg Test, Dominant (s)	Nine-Hole Peg Test, Non-Dominant (s)	MIRS Score
20	Healthy	Male	50.44	46.97	2.20	2.41	2.91	15.51	16.46	
20	Healthy	Female	36.15	32.86	3.01	3.30	3.48	13.01	14.69	
20	DM	Male	25.85	24.43	6.74	8.43	7.43	25.40	25.26	2.75
20	DM	Female	11.56	10.32	7.55	9.32	7.99	22.89	23.49	2.80
30	Healthy	Male	47.57	44.39	2.63	2.93	3.16	16.72	17.79	
30	Healthy	Female	33.28	30.28	3.43	3.82	3.72	14.21	16.01	
30	DM	Male	22.98	21.85	7.17	8.95	7.68	26.60	26.58	3.09
30	DM	Female	8.69	7.74	7.97	9.84	8.24	24.10	24.81	3.14
40	Healthy	Male	44.69	41.80	3.05	3.46	3.40	17.93	19.11	
40	Healthy	Female	30.40	27.69	3.86	4.34	3.97	15.42	17.34	
40	DM	Male	20.10	19.26	7.59	9.48	7.92	27.81	27.91	3.44
40	DM	Female	5.81	5.15	8.40	10.36	8.49	25.31	26.14	3.48
50	Healthy	Male	41.82	39.21	3.48	3.98	3.65	19.13	20.43	
50	Healthy	Female	27.53	25.10	4.28	4.86	4.22	16.63	18.66	
50	DM	Male	17.23	16.67	8.02	10.00	8.17	29.02	29.23	3.78

50	DM	Female	2.94	2.56	8.82	10.88	8.74	26.51	27.46	3.82
60	Healthy	Male	38.95	36.63	3.91	4.50	3.90	20.34	21.76	
60	Healthy	Female	24.66	22.52	4.71	5.39	4.47	17.83	19.98	
60	DM	Male	14.36	14.09	8.44	10.52	8.42	30.22	30.55	4.12
60	DM	Female	0.07	0	9.25	11.41	8.98	27.72	28.78	4.16
70	Healthy	Male	36.08	34.04	4.33	5.03	4.15	21.55	23.08	
70	Healthy	Female	21.79	19.93	5.14	5.91	4.71	19.04	21.31	
70	DM	Male	11.49	11.50	8.87	11.05	8.67	31.43	31.88	4.46
70	DM	Female	0	0	9.67	11.93	9.23	28.93	30.11	4.51
80	Healthy	Male	33.20	31.45	4.76	5.55	4.39	22.75	24.40	
80	Healthy	Female	18.92	17.35	5.56	6.43	4.96	20.25	22.63	
80	DM	Male	8.61	8.91	9.30	11.57	8.91	32.64	33.20	4.80
80	DM	Female	0	0	10.10	12.45	9.48	30.13	31.43	4.85
90	Healthy	Male	30.33	28.87	5.18	6.07	4.64	23.96	25.73	
90	Healthy	Female	16.04	14.76	5.99	6.96	5.21	21.45	23.95	
90	DM	Male	5.74	6.33	9.72	12.09	9.16	33.85	34.52	5.14
90	DM	Female	0		10.53	12.98	9.73	31.34	32.75	5.19

Supplementary Table S2. Linear regression results for grip strength, dominant hand (kg).

Variable	Coefficient	95% CI, Lower	95% CI, Upper	Std. Error	t-value	p-value
Constant	56.1820	52.2869	60.0771	1.9728	28.478	<0.001
Age	-0.2872	-0.3568	-0.2177	0.0352	-8.152	<0.001
group1	-24.5897	-27.7462	-21.4333	1.5987	-15.381	<0.001
sex1	-14.2890	-16.7231	-11.8549	1.2328	-11.590	<0.001

Supplementary Table S3. Linear regression results for grip strength, non-dominant hand (kg).

Variable	Coefficient	95% CI, Lower	95% CI, Upper	Std. Error	t-value	p-value
Constant	52.1456	48.5773	55.7139	1.8073	28.852	<0.001
Age	-0.2586	-0.3224	-0.1949	0.0323	-8.013	<0.001
group1	-22.5405	-25.4322	-19.6489	1.4646	-15.390	<0.001
sex1	-14.1093	-16.3392	-11.8795	1.1294	-12.493	<0.001

Supplementary Table S4. Linear regression results for eye opening (s).

Variable	Coefficient	95% CI, Lower	95% CI, Upper	Std. Error	t-value	p-value
Constant	1.3518	0.1425	2.5612	0.6125	2.207	0.0287
Age	0.0426	0.0210	0.0642	0.0109	3.892	<0.001
group1	4.5385	3.5585	5.5186	0.4964	9.143	<0.001
sex1	0.8032	0.0475	1.5590	0.3828	2.098	0.0374

Supplementary Table S5. Linear regression results for palm opening (s).

Variable	Coefficient	95% CI, Lower	95% CI, Upper	Std. Error	t-value	p-value
Constant	1.3662	0.1473	2.5852	0.6174	2.213	0.0283
Age	0.0523	0.0305	0.0741	0.0110	4.742	<0.001
group1	6.0194	5.0316	7.0072	0.5003	12.031	<0.001
sex1	0.8842	0.1225	1.6460	0.3858	2.292	0.0232

Supplementary Table S6. Linear regression results for tongue extension (s).

Variable	Coefficient	95% CI, Lower	95% CI, Upper	Std. Error	t-value	p-value
Constant	2.4148	1.4419	3.3877	0.4927	4.901	<0.001
Age	0.0247	0.0074	0.0421	0.0088	2.811	0.0055
group1	4.5186	3.7302	5.3070	0.3993	11.316	<0.001
sex1	0.5667	-0.0413	1.1746	0.3079	1.840	0.0675

Supplementary Table S7. Linear regression results for nine-hole peg test, dominant hand (s).

Variable	Coefficient	95% CI, Lower	95% CI, Upper	Std. Error	t-value	p-value
Constant	13.0984	11.0276	15.1691	1.0468	12.513	<0.001
Age	0.1207	0.0738	0.1675	0.0237	5.094	<0.001
group1	9.8860	8.0920	11.6800	0.9069	10.901	<0.001
sex1	-2.5059	-3.9560	-1.0558	0.7330	-3.419	<0.001

Supplementary Table S8. Linear regression results for nine-hole peg test, non-dominant hand (s).

Variable	Coefficient	95% CI Lower	95% CI Upper	Std. Error	t-value	p-value
Constant	13.8160	11.7291	15.9030	1.0550	13.096	<0.001
Age	0.1323	0.0851	0.1796	0.0239	5.543	<0.001
group1	8.7979	6.9899	10.6060	0.9140	9.626	<0.001
sex1	-1.7717	-3.2332	-0.3103	0.7388	-2.398	0.0179

Supplementary Table S9. Linear regression results for MIRS (Muscular Impairment Rating Scale) score.

Variable	Coefficient	95% CI, Lower	95% CI, Upper	Std. Error	t-value	p-value
Constant	2.0691	0.8071	3.3310	0.6161	3.359	0.0023
Age	0.0342	0.0082	0.0602	0.0127	2.691	0.0119
sex1	0.0462	-0.6150	0.7075	0.3228	0.143	0.8871
